# 1-[4-(Iodo­meth­yl)cyclo­hex­yl]-4-methyl­benzene

**DOI:** 10.1107/S160053681101765X

**Published:** 2011-05-14

**Authors:** Juan Wang, Erhong Duan, Erpeng Zhou, Qingguo Yao, Wenna Zhang

**Affiliations:** a School of Chemical Engineering, Shijiazhuang University, Shijiazhuang 050035, People’s Republic of China; b School of Environmental Science and Engineering, Hebei University of Science and Technology, Shijiazhuang 050018, People’s Republic of China

## Abstract

In the title compound, C_14_H_19_I, the cyclo­hexane ring adopts a chair conformation and the substituents are in equatorial sites. The dihedral angle between the mean planes of the cyclo­hexane and benzene rings is 67.23 (13)°.

## Related literature

The title compound is an inter­mediate in the praparation of liquid crystals. For background to liquid crystals, see: Demus & Hauser (1990 ▶). For the synthesis, see: Kozhushkov *et al.* (2004 ▶).
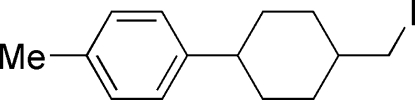


## Experimental

### 

#### Crystal data

C_14_H_19_I
*M* *_r_* = 314.19
Monoclinic, 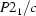

*a* = 17.593 (4) Å
*b* = 5.7722 (12) Å
*c* = 13.319 (3) Å
β = 105.71 (3)°
*V* = 1302.0 (5) Å^3^
*Z* = 4
Mo *K*α radiation
μ = 2.43 mm^−1^
*T* = 113 K
0.20 × 0.18 × 0.12 mm

#### Data collection

Rigaku Saturn CCD diffractometer
Absorption correction: multi-scan (*CrystalClear*; Rigaku/MSC, 2005 ▶) *T*_min_ = 0.642, *T*_max_ = 0.759
8233 measured reflections
2264 independent reflections
2077 reflections with *I* > 2σ(*I*)
*R*_int_ = 0.030

#### Refinement

*R*[*F* ^2^ > 2σ(*F* ^2^)] = 0.028
*wR*(*F* ^2^) = 0.074
*S* = 1.13
2264 reflections
137 parameters
H-atom parameters constrained
Δρ_max_ = 0.52 e Å^−3^
Δρ_min_ = −1.49 e Å^−3^

### 

Data collection: *CrystalClear* (Rigaku/MSC, 2005 ▶); cell refinement: *CrystalClear*; data reduction: *CrystalClear*; program(s) used to solve structure: *SHELXS97* (Sheldrick, 2008 ▶); program(s) used to refine structure: *SHELXL97* (Sheldrick, 2008 ▶); molecular graphics: *SHELXTL* (Sheldrick, 2008 ▶); software used to prepare material for publication: *CrystalStructure* (Rigaku/MSC, 2005 ▶).

## Supplementary Material

Crystal structure: contains datablocks I, global. DOI: 10.1107/S160053681101765X/hb5876sup1.cif

Structure factors: contains datablocks I. DOI: 10.1107/S160053681101765X/hb5876Isup2.hkl

Supplementary material file. DOI: 10.1107/S160053681101765X/hb5876Isup3.cml

Additional supplementary materials:  crystallographic information; 3D view; checkCIF report

## Figures and Tables

**Fig. 1.**
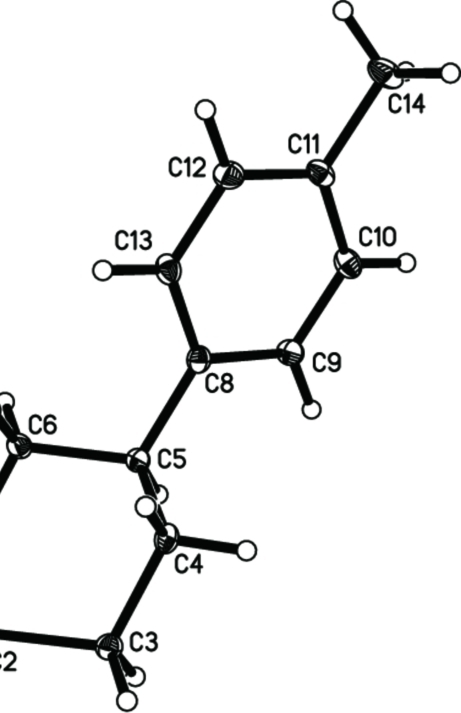
The molecular structure of (I) with 50% probability displacement ellipsoids.

## supplementary crystallographic information

### Comment

The title compound, (I), is an intermediate of liquid crystal compounds (Demus *et al.*,1990), which is synthesized from 1-bromo- 4-methylbenzene and 1-bromo-4-(iodomethyl)cyclohexane using diethyl ether as solvent (Kozhushkov *et al.*,2004). Herein, we report the title compound crystal structure.

In the molecule, Fig 1, a cyclohexane ring was attached at the *para* position of a benzene ring. The cyclohexyl ring has a typical chair conformation, with the tosion angles C3/C4/C5/C6 and C4/C5/C6/C7 being 56.7 (3) ° and -56.4 (3)°. No significant H-bonding or stacking interactions occur in the molecule packing.

### Experimental

1-(4-(Iodomethyl)cyclohexyl)-4-methylbenzene was synthesized according to the method described by Kozhushkov *et al.* (2004). Colourless prisms of (I) were obtained by evaporation from its ethanoic solution at room temperature (m.p. 318–319 K).

### Refinement

All H atoms were positioned geometrically and constrained to ride on their parent atoms [C—H distances are 0.95, 0.99 and 1.0Å with *U*_iso_(H) = 1.2 *U*_eq_(C) for aromatic and other aliphatic atoms, 0.98Å with *U*_iso_ = 1.5*U*_eq_ (C) for CH_3_ atoms].

### Crystal data

**Table:**

C_14_H_19_I	*F*(000) = 624
*M**_r_* = 314.19	*D*_x_ = 1.603 Mg m^−^^3^
Monoclinic, *P*2_1_/*c*	Mo *K*α radiation, λ = 0.71073 Å
Hall symbol: -P 2ybc	Cell parameters from 4209 reflections
*a* = 17.593 (4) Å	θ = 2.4–27.9°
*b* = 5.7722 (12) Å	µ = 2.43 mm^−^^1^
*c* = 13.319 (3) Å	*T* = 113 K
β = 105.71 (3)°	Prism, colourless
*V* = 1302.0 (5) Å^3^	0.20 × 0.18 × 0.12 mm
*Z* = 4	

### Data collection

**Table:**

Rigaku Saturn CCD diffractometer	2264 independent reflections
Radiation source: rotating anode	2077 reflections with *I* > 2σ(*I*)
confocal	*R*_int_ = 0.030
Detector resolution: 7.31 pixels mm^-1^	θ_max_ = 25.0°, θ_min_ = 2.4°
ω and φ scans	*h* = −20→19
Absorption correction: multi-scan (*CrystalClear*; Rigaku/MSC, 2005)	*k* = −5→6
*T*_min_ = 0.642, *T*_max_ = 0.759	*l* = −15→15
8233 measured reflections	

### Refinement

**Table:**

Refinement on *F*^2^	Primary atom site location: structure-invariant direct methods
Least-squares matrix: full	Secondary atom site location: difference Fourier map
*R*[*F*^2^ > 2σ(*F*^2^)] = 0.028	Hydrogen site location: inferred from neighbouring sites
*wR*(*F*^2^) = 0.074	H-atom parameters constrained
*S* = 1.13	*w* = 1/[σ^2^(*F*_o_^2^) + (0.0482*P*)^2^] where *P* = (*F*_o_^2^ + 2*F*_c_^2^)/3
2264 reflections	(Δ/σ)_max_ = 0.002
137 parameters	Δρ_max_ = 0.52 e Å^−^^3^
0 restraints	Δρ_min_ = −1.49 e Å^−^^3^

### Special details

**Table:**

Geometry. All e.s.d.'s (except the e.s.d. in the dihedral angle between two l.s. planes) are estimated using the full covariance matrix. The cell e.s.d.'s are taken into account individually in the estimation of e.s.d.'s in distances, angles and torsion angles; correlations between e.s.d.'s in cell parameters are only used when they are defined by crystal symmetry. An approximate (isotropic) treatment of cell e.s.d.'s is used for estimating e.s.d.'s involving l.s. planes.
Refinement. Refinement of *F*^2^ against ALL reflections. The weighted *R*-factor *wR* and goodness of fit *S* are based on *F*^2^, conventional *R*-factors *R* are based on *F*, with *F* set to zero for negative *F*^2^. The threshold expression of *F*^2^ > σ(*F*^2^) is used only for calculating *R*-factors(gt) *etc*. and is not relevant to the choice of reflections for refinement. *R*-factors based on *F*^2^ are statistically about twice as large as those based on *F*, and *R*- factors based on ALL data will be even larger.

### Fractional atomic coordinates and isotropic or equivalent isotropic displacement parameters (Å^2^)

**Table:**

	*x*	*y*	*z*	*U*_iso_*/*U*_eq_	
I1	0.069728 (10)	1.27167 (3)	0.586560 (14)	0.02224 (11)	
C1	0.08795 (15)	0.9476 (4)	0.6716 (2)	0.0203 (6)	
H1A	0.0358	0.8786	0.6681	0.024*	
H1B	0.1162	0.8396	0.6364	0.024*	
C2	0.13409 (14)	0.9681 (4)	0.78479 (19)	0.0141 (5)	
H2	0.1054	1.0764	0.8205	0.017*	
C3	0.13670 (19)	0.7272 (4)	0.8348 (2)	0.0189 (6)	
H3A	0.0821	0.6747	0.8291	0.023*	
H3B	0.1610	0.6158	0.7961	0.023*	
C4	0.18367 (18)	0.7284 (4)	0.9493 (2)	0.0182 (6)	
H4A	0.1856	0.5694	0.9777	0.022*	
H4B	0.1567	0.8288	0.9892	0.022*	
C5	0.26817 (15)	0.8169 (4)	0.9629 (2)	0.0153 (5)	
H5	0.2931	0.7121	0.9211	0.018*	
C6	0.26524 (15)	1.0592 (4)	0.9148 (2)	0.0192 (6)	
H6A	0.3197	1.1131	0.9206	0.023*	
H6B	0.2407	1.1686	0.9540	0.023*	
C7	0.21762 (14)	1.0587 (4)	0.79919 (19)	0.0183 (6)	
H7A	0.2152	1.2184	0.7713	0.022*	
H7B	0.2450	0.9606	0.7589	0.022*	
C8	0.31731 (17)	0.8004 (4)	1.0745 (2)	0.0176 (6)	
C9	0.36274 (15)	0.6031 (4)	1.1085 (2)	0.0196 (6)	
H9	0.3623	0.4812	1.0605	0.024*	
C10	0.40846 (15)	0.5803 (5)	1.2105 (2)	0.0219 (6)	
H10	0.4389	0.4438	1.2307	0.026*	
C11	0.41059 (18)	0.7517 (4)	1.2830 (3)	0.0202 (7)	
C12	0.36493 (16)	0.9486 (4)	1.2509 (2)	0.0236 (6)	
H12	0.3654	1.0694	1.2995	0.028*	
C13	0.31856 (15)	0.9713 (4)	1.1484 (2)	0.0213 (6)	
H13	0.2872	1.1063	1.1287	0.026*	
C14	0.4619 (2)	0.7254 (5)	1.3942 (3)	0.0281 (7)	
H14A	0.4348	0.6273	1.4337	0.042*	
H14B	0.4719	0.8782	1.4272	0.042*	
H14C	0.5123	0.6535	1.3935	0.042*	

### Atomic displacement parameters (Å^2^)

**Table:**

	*U*^11^	*U*^22^	*U*^33^	*U*^12^	*U*^13^	*U*^23^
I1	0.02506 (16)	0.01680 (15)	0.02078 (17)	−0.00064 (6)	−0.00077 (11)	0.00342 (6)
C1	0.0228 (15)	0.0154 (13)	0.0209 (15)	−0.0007 (11)	0.0028 (12)	0.0017 (11)
C2	0.0136 (13)	0.0133 (13)	0.0153 (14)	0.0027 (9)	0.0037 (11)	−0.0018 (10)
C3	0.0193 (16)	0.0200 (14)	0.0175 (17)	−0.0035 (10)	0.0053 (13)	0.0004 (10)
C4	0.0207 (16)	0.0166 (14)	0.0201 (17)	−0.0014 (10)	0.0100 (13)	0.0026 (10)
C5	0.0156 (14)	0.0148 (12)	0.0155 (14)	0.0024 (10)	0.0044 (11)	−0.0013 (10)
C6	0.0158 (14)	0.0206 (14)	0.0191 (15)	−0.0035 (11)	0.0012 (11)	0.0038 (11)
C7	0.0175 (14)	0.0179 (13)	0.0193 (15)	−0.0023 (10)	0.0044 (11)	0.0045 (10)
C8	0.0168 (15)	0.0171 (13)	0.0198 (16)	−0.0007 (10)	0.0067 (12)	0.0019 (11)
C9	0.0193 (15)	0.0191 (14)	0.0197 (15)	0.0047 (11)	0.0038 (12)	−0.0021 (11)
C10	0.0192 (15)	0.0253 (14)	0.0212 (16)	0.0062 (11)	0.0051 (12)	0.0035 (11)
C11	0.0167 (16)	0.0292 (16)	0.0153 (17)	−0.0044 (10)	0.0050 (13)	0.0038 (10)
C12	0.0293 (16)	0.0245 (14)	0.0187 (16)	−0.0012 (12)	0.0092 (13)	−0.0032 (11)
C13	0.0267 (16)	0.0186 (13)	0.0194 (15)	0.0071 (11)	0.0079 (12)	0.0020 (11)
C14	0.0184 (17)	0.046 (2)	0.0187 (18)	−0.0042 (12)	0.0027 (14)	0.0033 (12)

### Geometric parameters (Å, °)

**Table:**

I1—C1	2.165 (3)	C6—H6B	0.9900
C1—C2	1.511 (3)	C7—H7A	0.9900
C1—H1A	0.9900	C7—H7B	0.9900
C1—H1B	0.9900	C8—C13	1.389 (4)
C2—C7	1.522 (3)	C8—C9	1.394 (3)
C2—C3	1.537 (3)	C9—C10	1.385 (4)
C2—H2	1.0000	C9—H9	0.9500
C3—C4	1.525 (4)	C10—C11	1.376 (4)
C3—H3A	0.9900	C10—H10	0.9500
C3—H3B	0.9900	C11—C12	1.391 (4)
C4—C5	1.536 (4)	C11—C14	1.519 (5)
C4—H4A	0.9900	C12—C13	1.393 (4)
C4—H4B	0.9900	C12—H12	0.9500
C5—C8	1.507 (4)	C13—H13	0.9500
C5—C6	1.533 (3)	C14—H14A	0.9800
C5—H5	1.0000	C14—H14B	0.9800
C6—C7	1.541 (3)	C14—H14C	0.9800
C6—H6A	0.9900		
			
C2—C1—I1	114.67 (17)	C5—C6—H6B	109.4
C2—C1—H1A	108.6	C7—C6—H6B	109.4
I1—C1—H1A	108.6	H6A—C6—H6B	108.0
C2—C1—H1B	108.6	C2—C7—C6	111.81 (19)
I1—C1—H1B	108.6	C2—C7—H7A	109.3
H1A—C1—H1B	107.6	C6—C7—H7A	109.3
C1—C2—C7	113.1 (2)	C2—C7—H7B	109.3
C1—C2—C3	107.8 (2)	C6—C7—H7B	109.3
C7—C2—C3	110.0 (2)	H7A—C7—H7B	107.9
C1—C2—H2	108.6	C13—C8—C9	116.9 (3)
C7—C2—H2	108.6	C13—C8—C5	123.3 (2)
C3—C2—H2	108.6	C9—C8—C5	119.8 (2)
C4—C3—C2	111.9 (2)	C10—C9—C8	121.7 (2)
C4—C3—H3A	109.2	C10—C9—H9	119.1
C2—C3—H3A	109.2	C8—C9—H9	119.1
C4—C3—H3B	109.2	C11—C10—C9	121.2 (2)
C2—C3—H3B	109.2	C11—C10—H10	119.4
H3A—C3—H3B	107.9	C9—C10—H10	119.4
C3—C4—C5	111.4 (2)	C10—C11—C12	117.9 (3)
C3—C4—H4A	109.3	C10—C11—C14	120.6 (2)
C5—C4—H4A	109.3	C12—C11—C14	121.6 (2)
C3—C4—H4B	109.3	C11—C12—C13	121.0 (3)
C5—C4—H4B	109.3	C11—C12—H12	119.5
H4A—C4—H4B	108.0	C13—C12—H12	119.5
C8—C5—C6	114.5 (2)	C8—C13—C12	121.3 (2)
C8—C5—C4	112.0 (2)	C8—C13—H13	119.4
C6—C5—C4	109.4 (2)	C12—C13—H13	119.4
C8—C5—H5	106.8	C11—C14—H14A	109.5
C6—C5—H5	106.8	C11—C14—H14B	109.5
C4—C5—H5	106.8	H14A—C14—H14B	109.5
C5—C6—C7	111.3 (2)	C11—C14—H14C	109.5
C5—C6—H6A	109.4	H14A—C14—H14C	109.5
C7—C6—H6A	109.4	H14B—C14—H14C	109.5
			
I1—C1—C2—C7	−61.9 (2)	C4—C5—C8—C13	−85.8 (3)
I1—C1—C2—C3	176.26 (17)	C6—C5—C8—C9	−142.4 (2)
C1—C2—C3—C4	178.9 (2)	C4—C5—C8—C9	92.3 (3)
C7—C2—C3—C4	55.1 (3)	C13—C8—C9—C10	−1.5 (4)
C2—C3—C4—C5	−56.9 (3)	C5—C8—C9—C10	−179.7 (2)
C3—C4—C5—C8	−175.25 (19)	C8—C9—C10—C11	0.4 (4)
C3—C4—C5—C6	56.7 (3)	C9—C10—C11—C12	0.3 (4)
C8—C5—C6—C7	177.0 (2)	C9—C10—C11—C14	−179.2 (2)
C4—C5—C6—C7	−56.4 (3)	C10—C11—C12—C13	0.0 (4)
C1—C2—C7—C6	−175.4 (2)	C14—C11—C12—C13	179.5 (2)
C3—C2—C7—C6	−54.8 (3)	C9—C8—C13—C12	1.8 (4)
C5—C6—C7—C2	56.8 (3)	C5—C8—C13—C12	180.0 (2)
C6—C5—C8—C13	39.4 (3)	C11—C12—C13—C8	−1.1 (4)
